# Comprehensive analysis of bacteriocins in *Streptococcus mutans*

**DOI:** 10.1038/s41598-021-92370-1

**Published:** 2021-06-21

**Authors:** Atsuko Watanabe, Miki Kawada-Matsuo, Mi Nguyen-Tra Le, Junzo Hisatsune, Yuichi Oogai, Yoshio Nakano, Masanobu Nakata, Shouichi Miyawaki, Motoyuki Sugai, Hitoshi Komatsuzawa

**Affiliations:** 1 Department of Orthodontics and Dentofacial Orthopedics, Kagoshima University Graduate School of Medical and Dental Sciences, Kagoshima, Japan; 2 Department of Bacteriology, Hiroshima University Graduate School of Biomedical and Health Sciences, Kasumi 1-2-3, Hiroshima City, Hiroshima, 734-8551 Japan; 3 Project Research Centre for Nosocomial Infectious Diseases, Hiroshima University, Hiroshima, Japan; 4 Antimicrobial Resistance Research Centre, National Institute of Infectious Diseases, Higashi Murayama, Japan; 5 Department of Oral Microbiology, Kagoshima University Graduate School of Medical and Dental Sciences, Kagoshima, Japan; 6 Department of Chemistry, Nihon University School of Dentistry, Tokyo, Japan

**Keywords:** Bacteriology, Microbial communities

## Abstract

*Streptococcus mutans* produces bacteriocins that show antibacterial activity against several bacteria. However, comprehensive analysis of these bacteriocins has not been well done. In this study, we isolated 125 *S. mutans* strains from volunteers and determined their whole genome sequence. Based on the genome analysis, the distribution of each bacteriocin gene (mutacins I-IV, K8 and Smb) was investigated. We found 17, 5, and 2 strains showing 100% matches with mutacin I, mutacin II and mutacin III, respectively. Five mutacin III-positive strains had 2 mismatches compared to mature mutacin III. In 67 mutacin IV-positive strains, 38 strains showed 100% match with mutacin IV, while 29 strains showed some variations. In 23 mutacin K8- and 32 mutacin Smb-positive strains, all except one mutacin K8-positive strain showed 100% match with the mature peptides. Among 125 strains, 84 (65.1%), 26 (20.2%), and 5 (3.9%) strains were positive for one, two and three bacteriocin genes, respectively. Then, the antibacterial activity against oral streptococci and other oral bacterial species was investigated by using bacteriocin gene single-positive strains. Each bacteriocin gene-positive strain showed a different pattern of antibacterial activity. These results speculate that individual *S. mutans* strains may affect the bacterial composition of dental plaques.

## Introduction

*Streptococcus mutans* is a commensal bacterium in the oral cavity. *S. mutans* is known to be a major cariogenic bacterium, especially for smooth surface caries, because *S. mutans* produces glucosyltransferases, which mediate the synthesis of exopolysaccharides called glucans, which are essential for dental plaque formation and produce acids for the demineralization of teeth^1–3^. In dental plaques, several hundred bacterial species are colocalized. Oral streptococci such as *S. mitis*, *S. oralis* and *S. salivarius* are early colonizers of the tooth surface^3,4^. *S. mutans* significantly contributes to the formation of an initial dental plaque. *S. mutans* has the ability to attach pellicle coatings onto teeth by PAc and sticky glucan^1,2^. This sticky glucan mediates the binding of other oral bacteria, promoting biofilm development on the tooth surface. Therefore, *S. mutans* interacts with many oral bacterial species, evolving mechanisms that allow it to compete or cooperate with other oral bacteria in dental plaques.

It is well known that many bacteria produce antibacterial peptides named bacteriocins^5–7^. Bacteriocins are ribosomally synthesized peptides or proteins. Generally, bacteriocins exhibit antibacterial activity against species that are closely related to bacteriocin producers, although some bacteriocins, such as nisin A produced by *Lactococcus lactis,* show a broad spectrum^8^. Bacteriocins are mainly classified into class I and class II groups^9,10^. Class I bacteriocins are known as lantibiotics that contain a ring bridged by unusual amino acids, lanthionine and 3-methyllanthionine residues. Class II bacteriocins are synthesized by unmodified amino acids. Lantibiotics are subdivided into A and B types^9,11^. Type A lantibiotics disturb the bacterial membrane, while type B lantibiotics are globular peptides that inhibit cell wall biosynthesis steps such as transglycosylation. Type A lantibiotics are further classified into two subtypes: type A(I), which includes nisin and epidermin, and type A(II), which includes lacticin 481 and nukacin ISK-1. Class II bacteriocins are classified into the following three subclasses: IIa, IIb, and IIc.

It has been reported that *S. mutans* produces several types of bacteriocins^12–19^. The major bacteriocins of *S. mutans* are mutacins I-IV, K8 and Smb. These bacteriocins were reported to have antibacterial activity against several bacterial species. These peptides, except mutacin IV, are lantibiotics. Mutacin IV is a two-component peptide (nlmA and nlmB) categorized in class II. Mutacin IV expression is regulated by ComDE^19^, which is a key factor for the quorum sensing system. Additionally, ComDE is responsible for genetic competence by regulating the expression of mutacin IV and ComYA-I (DNA uptake). Therefore, mutacin IV is reported to be involved in competence. In addition, mutacins V and VI were also reported as nonlantibiotic bacteriocins and are regulated by ComDE^19,20^. Although many investigations regarding the characterization of each bacteriocin have been performed, there are few studies reporting comparative analysis of the bacteriocins in *S. mutans*^21^.

In this study, we isolated 125 *S. mutans* strains from the oral cavity. We performed genomic analysis of all strains and characterized the genes coding for bacteriocins. Then, we evaluated the antibacterial activity of the strains against several oral bacteria. This is the first report of the comprehensive analysis of bacteriocins using a genome analysis approach.

## Results

### Distribution of bacteriocin genes among 125 *S. mutans* strains

**Figure 1:**
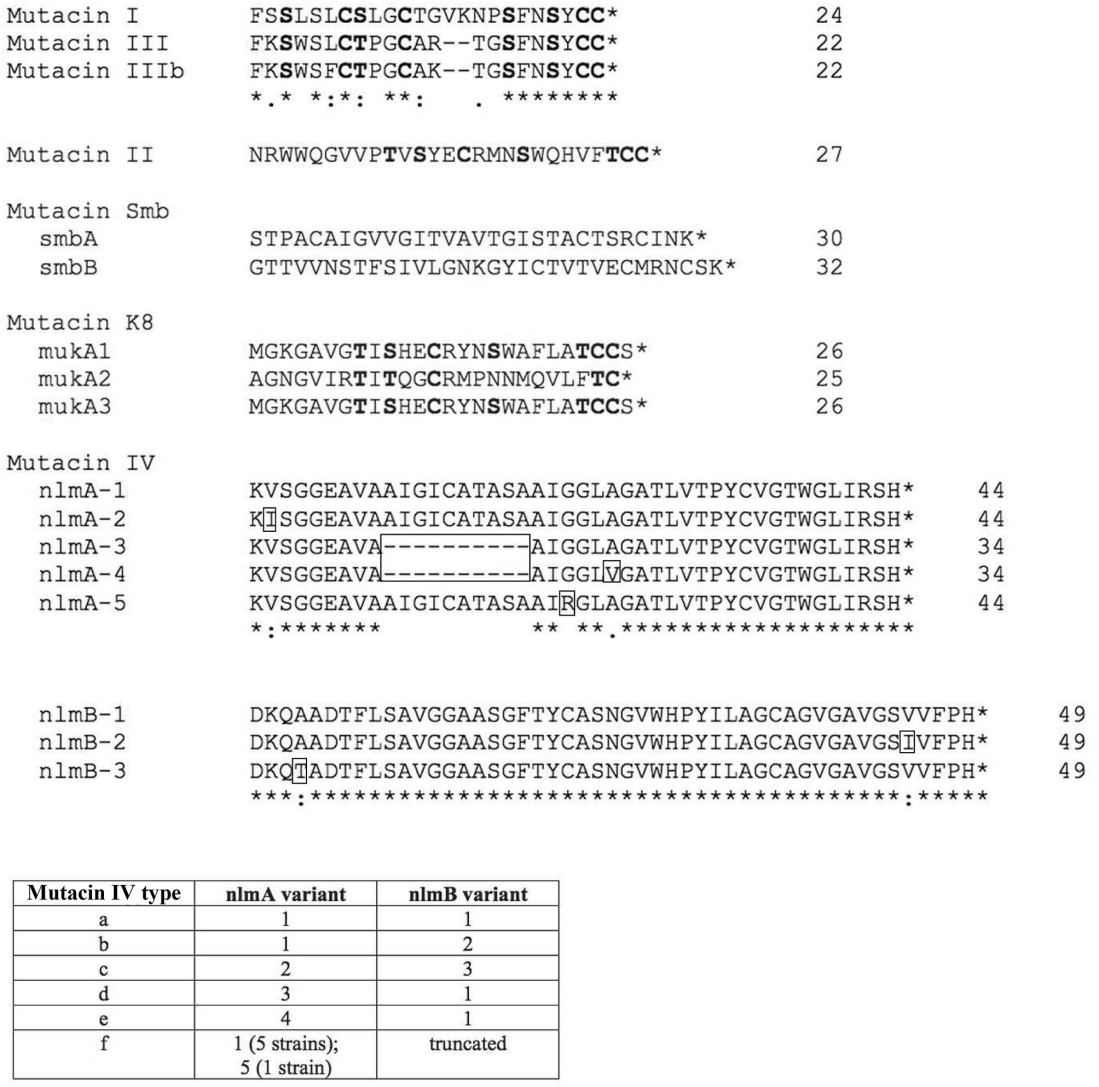
Amino acid sequence of the mature peptide of bacteriocin. Based on the genome sequence, the amino acid sequence of bacteriocin was determined. The bold font represents residues to be posttranslationally modified. The square represents a different amino acid from that of the reference peptide. Mutacin IV variants (a–f) were determined by the combination of nlmA and nlmB. Asterisk (*) indicates positions which have a single, fully conserved residue. Colon (:) indicates conservation between groups of strongly similar properties—scoring > 0.5 in the Gonnet PAM 250 matrix. Period (.) indicates conservation between groups of weakly similar properties—scoring =  < 0.5 in the Gonnet PAM 250 matrix.

Based on the genome sequence of each strain, the genes coding for mutacins I to IV, K8 and Smb were identified. The reference genes of each bacteriocin examined in this study were *mutA-I* (Accession No. AF207710), *mutA-II* (U40620), *mutA-III* (AF154675), *nlmAB* (NC_004350*), mukA1mukA3* (EF060238) and *smbAsmbB* (AB179778). Of 125 *S. mutans* strains, 115 strains possessed a bacteriocin (Table 1). Several bacteriocins, including mutacin III, mutacin IV and mutacin K8, had several variants compared to each reference gene at the nucleotide and amino acid levels (Table 2). We found 17, 5, and 2 strains showing 100% matches in nucleotide and amino acid levels with the *mutA* genes of mutacin I, mutacin II and mutacin III, respectively (Table 2). Five strains had a *mutA* gene of mutacin III with 11 mismatches in nucleotide sequence compared to the reference gene, encoding a peptide with 2 mismatches compared to the core mutacin III. Therefore, this 2-mismatch peptide was designated mutacin IIIb (Fig. 1). In contrast, no variations were found in the mutacin I and II genes. Among the mutacin IV-positive strains, 38/67 strains had the same *nlmAB* gene as the reference genes, while 29 strains showed some variations, which could be classified into 9 types of *nlmAB* regions at the nucleotide level. These variations resulted in 7 variants of nlmA and 3 variants of nlmB, including the reference peptide in the precursor peptide (Supplemental Fig. 1). In the core peptides of nlmA and nlmB, 4 variants of nlmA and 3 variants of nlmB were found (Fig. 1). Based on the mature peptides of nlmA and nlmB, we classified mutacin IV into mutacins IVa to IVf (Fig. 1). The *nlmB* gene of mutacin IVf was disrupted by the mutation. Among 23 mutacin K8-positive strains, 19 strains showed one nucleotide difference with the *mukA1* of the reference gene, but all showed 100% match with the reference mukA1 and mukA3 peptides. One mutacin K8-positive strain (KSM55) showed the loss of *mukA1* but has the intact *mukA3*. All mutacin Smb-positive strains showed one nucleotide difference with the *smbA* of the reference gene, but all showed 100% match with the reference SmbA and SmbB peptides.

**Table 1:** The number of strains by the type of the retention with bacteriocin genes.

	Mutacin I	Mutacin II	Mutacin III	Mutacin IV	Mutacin Smb	Mutacin K8	Total
Single positive	15	3	3	39	9	15	84
**Double positive**							
Mutacin IV +	–	–	3	–	17	3	23
Mutacin I +	–	2	–	–	–	–	2
Mutacin K8 +	–	–	–	–	1	–	1
**Triple positive mutacin**							
IV + Smb +	–	–	1	–	–	4	5
None							10

**Table 2:** The number of strains with mutations in nucleotide and peptide level.

	Total	Nucleotide		Premature peptide		Mature peptide	
		No	Mutations	No	Mutations	No	Mutations
Mutacin I	17	17	0	17	0	17	0
Mutacin II	5	5	0	5	0	5	0
Mutacin III	7	2	5	2	5	2	5
Mutacin IV	67	38	29	39	28	57	10
Mutacin Smb	32	0	32	0	32	32	0
Mutacin K8	23	3	20	22	1	22	1

The number of bacteriocin genes in each strain is shown in Table 1. Among 125 strains, 84 (65.1%), 26 (20.2%), and 5 (3.9%) strains were positive for one, two, and three genes, respectively. Among 84 single-positive strains, the number of mutacin IV-positive strains was the highest (39 strains, 46.4%). Among 26 double-positive strains, the number of strains with mutacin IV positivity was 23 (88.5%). In particular, 17 strains were mutacin IV- and Smb-positive (65.4%). Among the triple-positive strains, 4 strains were mutacin IV-, mutacin Smb- and mutacin K8-positive, and one strain was mutacin IV-, mutacin Smb- and mutacin III-positive.

### Phylogenetic analysis

**Figure 2:**
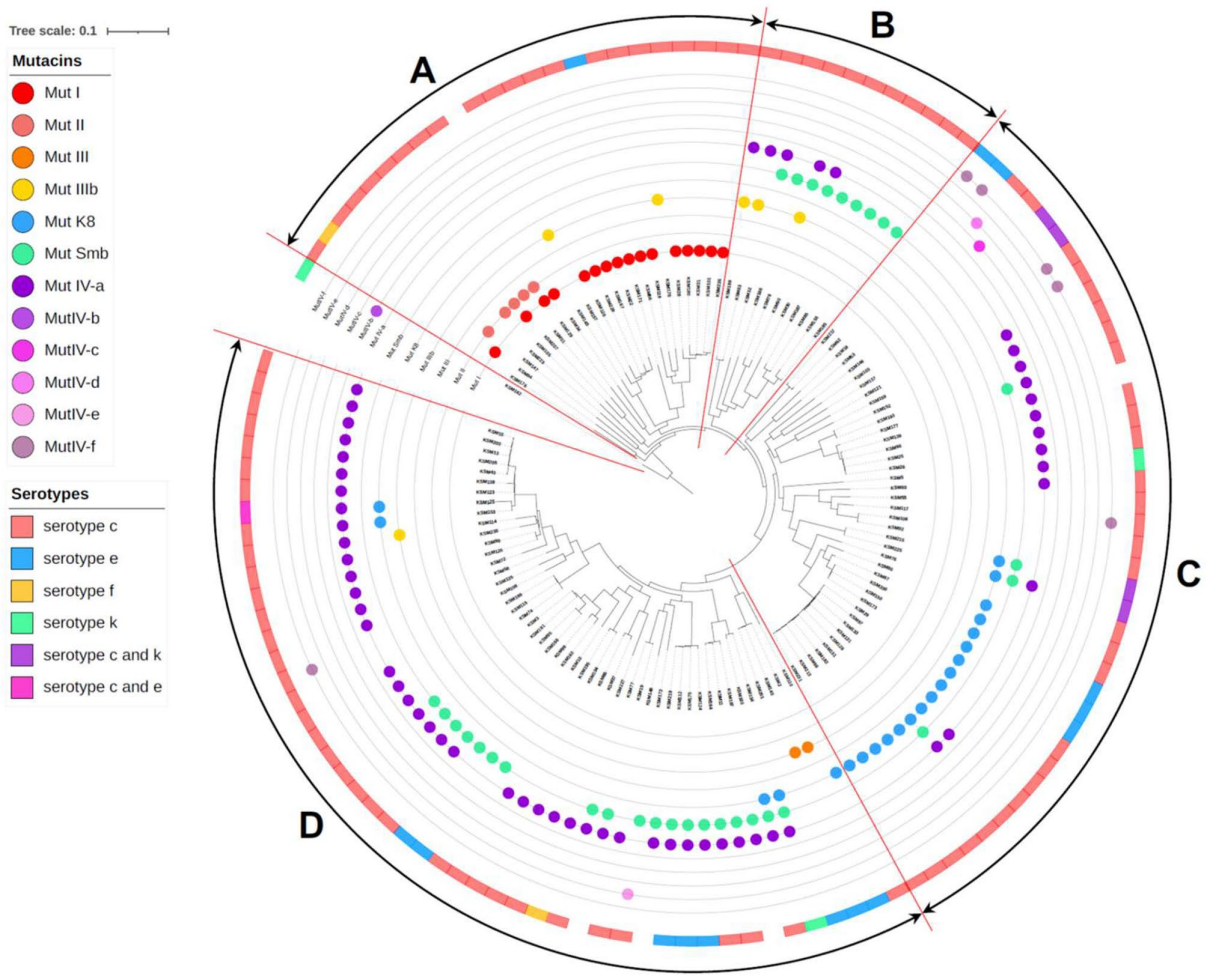
Phylogenetic tree of 125 *S. mutans* strains. A phylogenetic tree was constructed by the method described in the Materials and methods section using iTOL (https://itol.embl.de).

The SNP-based phylogenetic tree generated 4 main clusters (groups A, B, C and D) (Fig. 2). Interestingly, all mutacin I- or mutacin II-positive strains were classified into the same cluster (group A), while mutacin IV-positive strains were distributed in the remaining groups (group B, C and D). Mutacin IIIb-positive strains were distributed in three groups (group A, B and D), while mutacin III-positive strains were distributed in one group (group D). Eighteen mutacin K8-positive strains (78.3%) were classified into one group (group C), and mutacin Smb-positive strains were mainly classified into 2 groups (group B [9 strains] and D [19 strains]), with some sporadic Smb-positive strains belonging to group C. We also analysed the relationship between serotype antigens and bacteriocins. Most *S. mutans* strains showed serotype c, and a relationship between bacteriocin and serotype antigen was not found.

### Antibacterial activity of bacteriocins against oral bacteria

**Figure 3:**
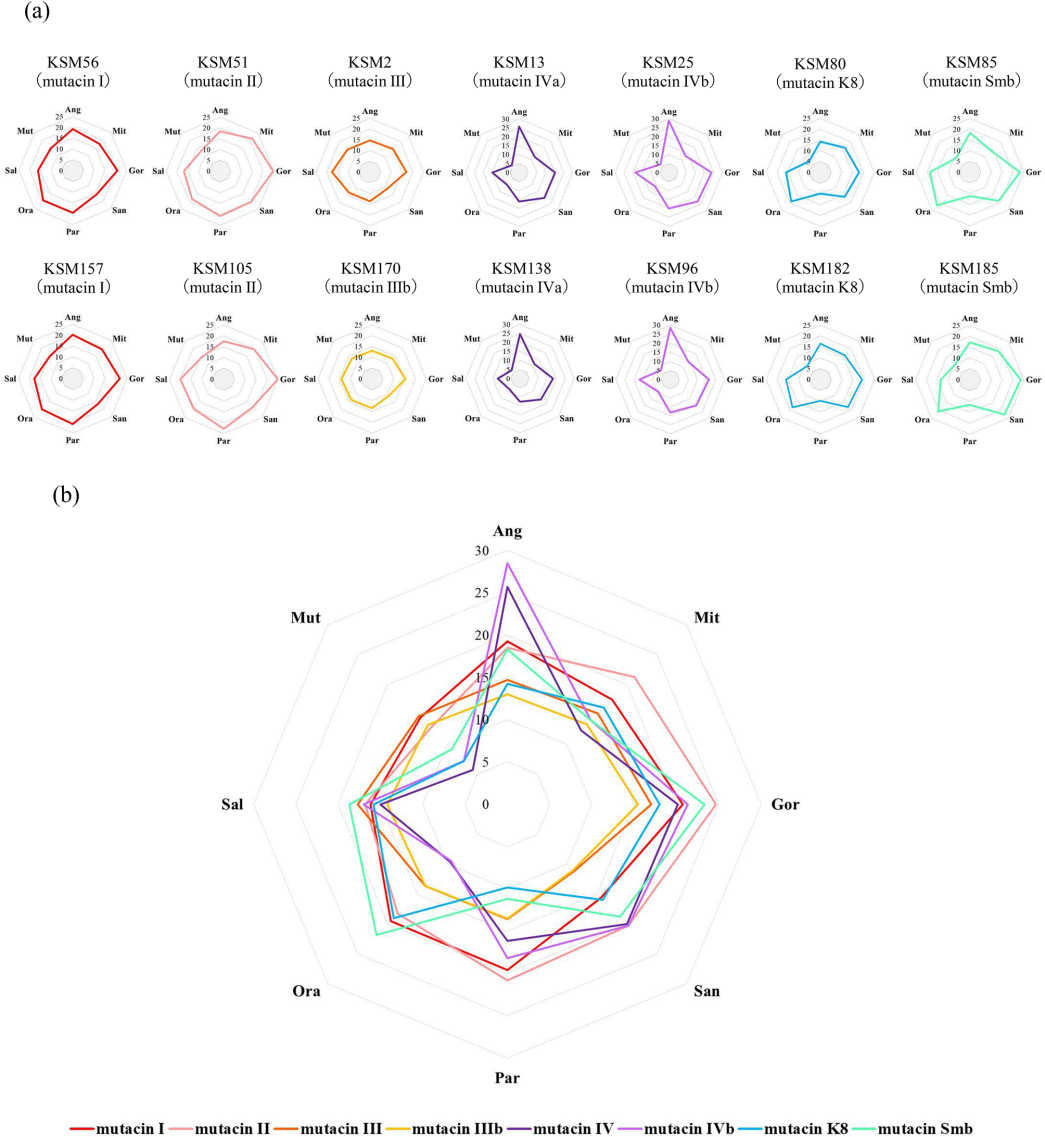
Antibacterial activity of each bacteriocin gene single-positive strain against 8 oral streptococcal species. To evaluate the antibacterial activity of bacteriocins, a direct assay was performed with the method described in the Materials and methods section. (**a**) Two single-positive strains for each bacteriocin except mutacin III and mutacin IIIb were selected. (**b**) Merged antibacterial activity of each single positive strain (KSM2, 13, 25, 56, 51, 80, 85, 170). Three independent experiments were performed, and the average diameter was calculated. The number in each panel represents the diameter of the inhibition zone (mm). Ang: *S. anginosus*, Mit: *S. mitis*, Gor: *S. gordonii*, San: *S. sanguinis*, Par: *S. parasanguinis*, Ora: *S. oralis*, Sal: *S. salivarius*, Mut: *S. mutans.*

**Figure 4:**
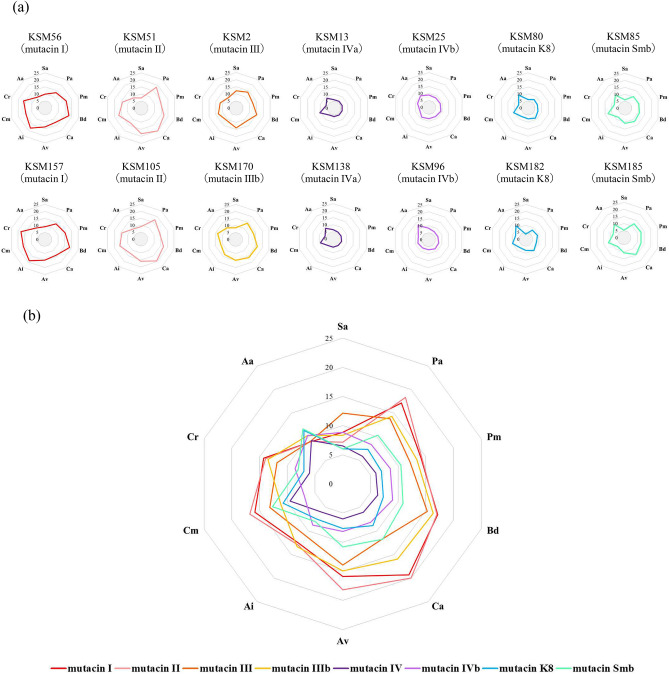
Antibacterial activity of each bacteriocin gene single-positive strain against 10 oral bacterial species. To evaluate the antibacterial activity of bacteriocins, a direct assay was performed with the method described in the Materials and methods section. (**a**) Two single-positive strains for each bacteriocin except mutacin III and mutacin IIIb were selected. (**b**) Merged antibacterial activity of each single positive strain (KSM2, 13, 25, 56, 51, 80, 85, 170). Three independent experiments were performed, and the average diameter was calculated. The number in each panel represents the diameter of the inhibition zone (mm). Sa: *S. aureus,* Pa: *P. anaerobius,* Pm: *P. micra*, Bd: *B. dentium*, Ca: *C. acnes*, Av: *A. viscosus,* Ai: *A. israelii,* Cm: *C. matruchotii*, Cr: *C. rectus*, Aa: *A. actinomycetemcomitans.*

Two single-positive strains for each bacteriocin except mutacin III (one strain) and mutacin IIIb (one strain) were selected and used for the evaluation of antibacterial activity against 8 oral streptococcal strains and 10 nonstreptococcal oral bacterial strains. Each strain showed a characteristic antibacterial pattern against oral bacteria depending on the bacteriocin (Figs. 3, 4). These antibacterial patterns were mainly divided into three groups by bacteriocin type. Mutacins I-, II-, III- and IIIb-positive strains showed broad activity against oral streptococci and other oral bacteria except *Aggregatibacter actinomycetemcomitans* and *Staphylococcus aureus*. Mutacin IVa- and IVb-positive strains showed strong activity against oral streptococci except *S. mitis* and *S. oralis* and less activity against other oral bacteria. Mutacin K8- and mutacin Smb-positive strains showed strong activity against oral streptococci except *S. parasanguinis* and weak activity against other oral bacteria.

**Figure 5:**
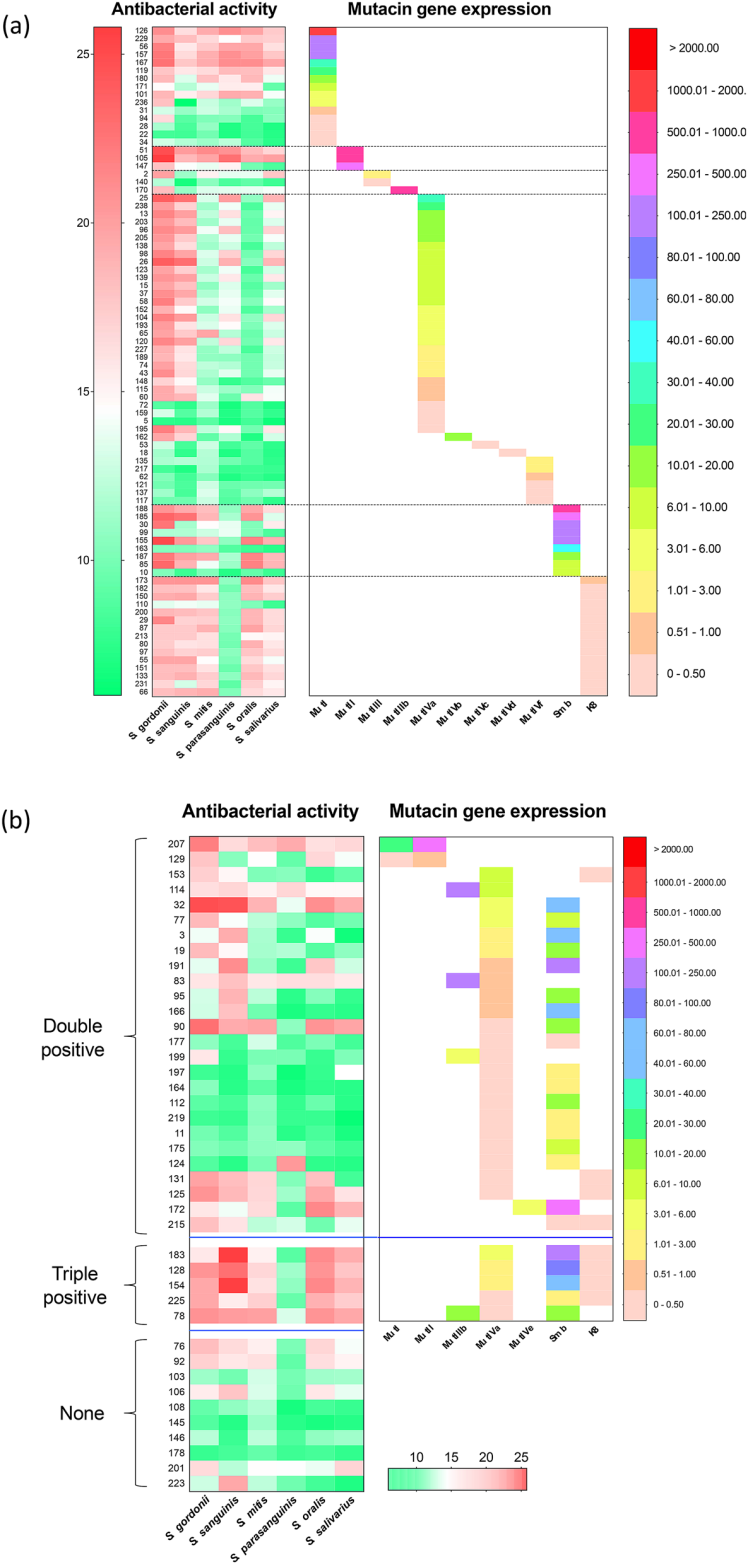
Heat map of antibacterial activity and gene expression in 125 *S. mutans* strains. Heat map was constructed by Graphpad Prism version 9.1.0 (https://www.graphpad.com). (**a**) Bacteriocin single-positive strains, (**b**) Bacteriocin double-positive strains, triple-positive strains and negative strains. The number represents the strain name. The colour scale on the left represents the antibacterial activity level (the diameter of the inhibitory zone), green represents low expression, and red represents high expression. The colour scale on the right represents the gene expression level (the ratio compared to *gyrA*), bisque represents low expression, and red represents high expression.

Then, we investigated the antibacterial activity of all strains against 6 oral streptococcal species (Fig. 5). In single positive strains, the pattern of antibacterial activity for the same bacteriocin type showed a similar tendency as the results shown in Fig. 3, although some strains showed weak antibacterial activity. Mutacin I (5 strains)-, mutacin III (1 strain)- and mutacin IV (11 strains)-positive strains, including 6 mutacin IVf-positive strains with the disrupted *nlmB* gene (KSM62, 117, 121, 135, 137, 217), showed very weak antibacterial activity. To compare the antibacterial activity with the gene expression, we performed quantitative PCR (qPCR) to evaluate the expression of each bacteriocin gene (Fig. 5a). The expression of the respective bacteriocin genes in the strains with weak antibacterial activity was significantly lower than those in the strains with high antibacterial activity.

**Figure 6:**
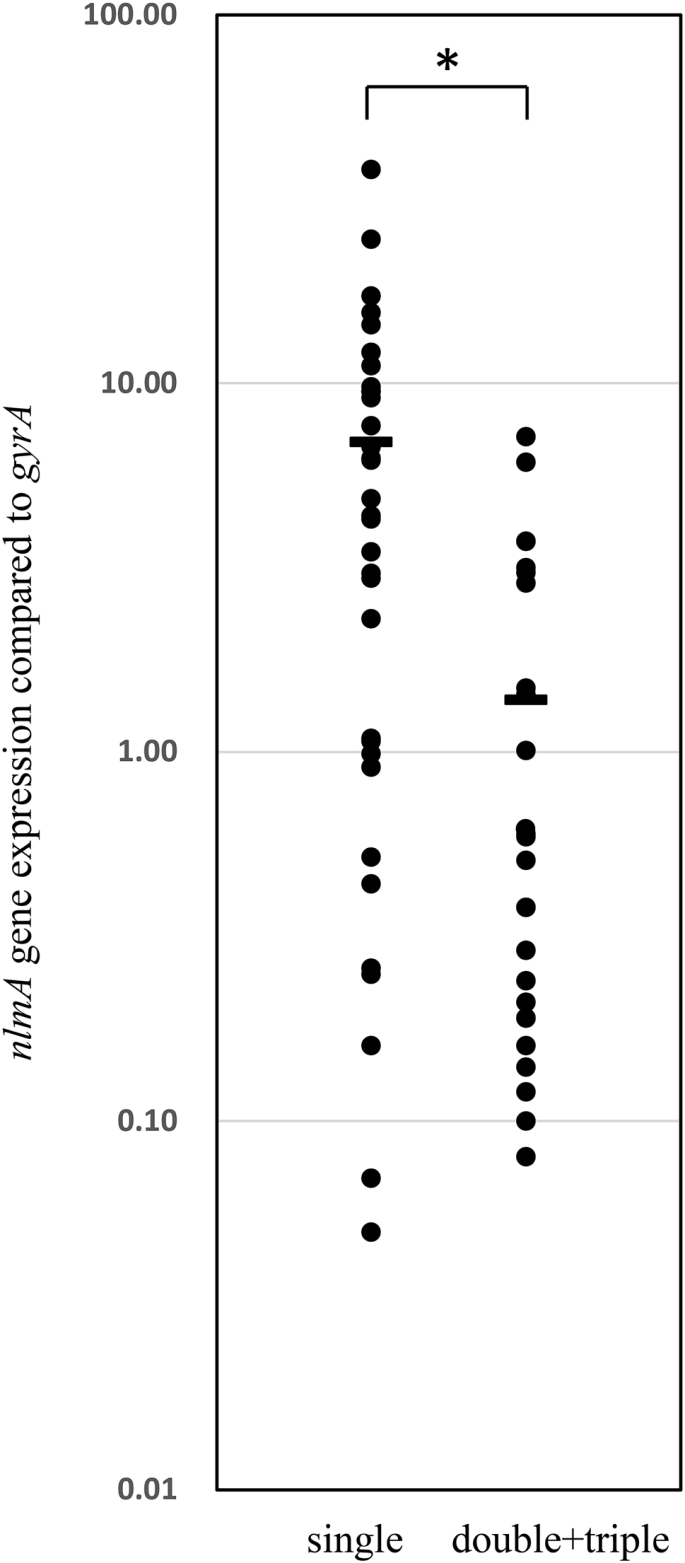
The *nlmA* gene expression in all mutacin IV-positive strains. NlmA gene expression in all mutacin IV-positive strains was analysed by the method described in the Materials and methods section. The horizontal bar indicates the average expression. *p < 0.05, as determined by *t* test.

Then, we investigated the antibacterial activity and the expression of each bacteriocin gene in double- and triple-bacteriocin-positive strains (Fig. 5b). Several double- and triple-bacteriocin-positive strains showed antibacterial activity, but the degree of antibacterial activity in these strains was not as high as that of single-positive strains due to the low expression of either bacteriocin gene. Since most strains were mutacin IV- positive, we compared mutacin IV gene expression among single-, double- and triple-positive strains (Fig. 6). The proportion of low-expression strains among the double- and triple-positive strains was greater than that among the single-positive strains.

### Comparison of the promoter and regulator regions of bacteriocin genes between strains with strong and weak antibacterial activity

**Figure 7:**
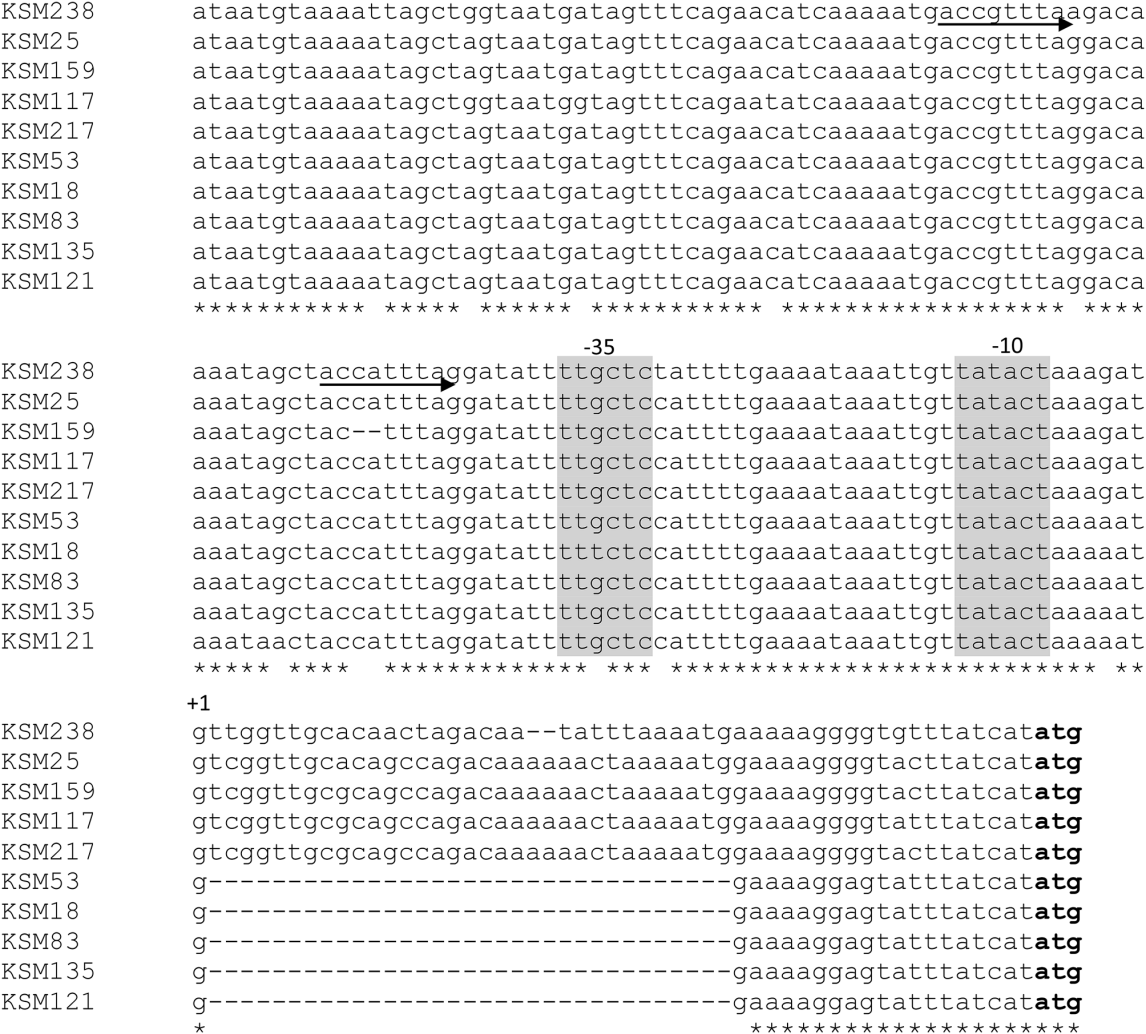
Comparison of the promoter region of mutacin IV gene between strains with strong and weak antibacterial activity. Nucleotide sequence upstream of *nlmA* in the strains with strong (KSM238 and KSM25) and weak antibacterial activity. The arrows represent the direct repeat sequences for the BrsR binding site. The bold letters represent the first codon of *nlmA*. Gray background represents the promoter region (− 35 and − 10 box).

By comparing the nucleotide sequences between strains with high and low antibacterial activity, we found several differences in the promoter regions and/or the regulator sequences. Among the 15 mutacin I-positive strains, all possessed the same promoter sequence upstream of *mutA*, while the strains with low levels of mutacin I expression (KSM22, 34, 94) showed some mutations in the *mutR* gene (mutacin operon transcription activator) (Supplemental Fig. 2). However, one strain (KSM28) with a low level of mutacin I expression showed similar sequences of the *mutA* and *mutR* regions compared with those of the high-expression strains. Among the 2 mutacin III-positive strains, comparison between the high (KSM2) and low (KSM140) *mutA*-expression strains also revealed a difference in the *mutR* sequence. The *mutR* gene in KSM140 was disrupted by one nucleotide insertion. For the mutacin IV -single positive isolates, the low *nlmAB* expression strains except mutacin IVf-positive strains (*nlmB* disrupted strains) showed some mutations in the promoter region upstream of *nlmA* (Fig. 7) or mutations in the *comCDE* region (KSM5, 72), which plays a role as a mutacin transcriptional regulator. Comparison of the promoter region upstream of *nlmA* between the high and low mutacin IV-expressing strains suggested the presence of a mutation (A to G) at nucleotide position + 10 relative to the *nlmA* transcription start site (TSS) in the strains with low levels of mutacin IV expression (KSM117, 217, 159) in comparison with those sequences in high expression strains (KSM25, 238) (Fig. 6). In addition, some other low-mutacin IV-expressing strains (KSM18, 53, 83, 121, 135) showed deletions in the nucleotide region from + 2 to + 34 relative to the *nlmA* TSS in comparison with the high-mutacin IV-expressing strains.

### Antibacterial activity of mutacin I-, V- and VI-KO mutants

**Figure 8:**
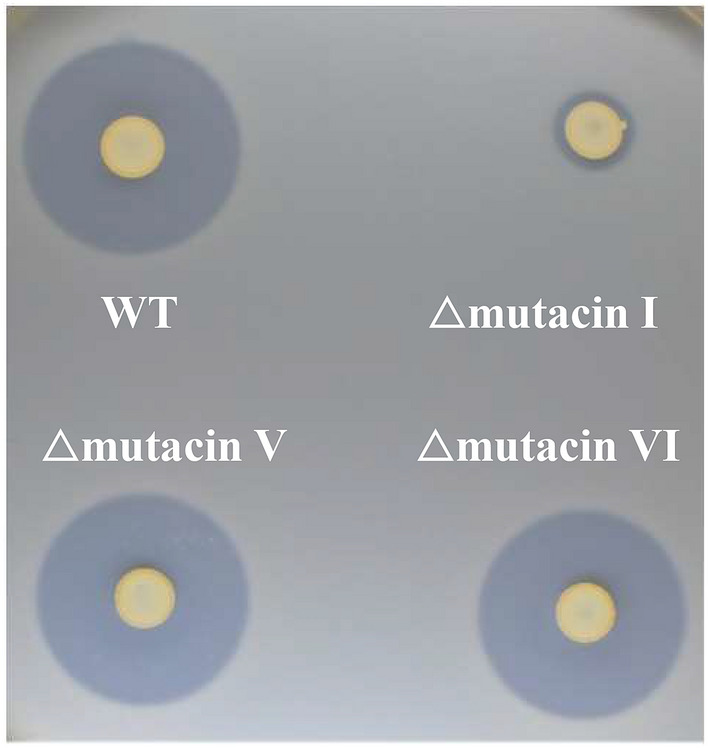
Antibacterial activity of mutacin I-, V- and VI-KO mutants. The antibacterial activity of the wild type (KSM157) and its mutants against *S. salivarius* was evaluated by the direct method described in the Materials and methods section.

To determine whether mutacins V and VI showed strong antibacterial activity, we tried to construct each KO mutant together with the mutacin I-KO mutant in KSM157 which were mutacins I-, V- and VI-positive. Compared to the WT strain (KSM157), the mutacin I-KO mutant showed a strong reduction in antibacterial activity, while the mutacin V- and mutacin VI-mutants showed a slight reduction in antibacterial activity (Fig. 8).

## Discussion

In this study, we performed the whole genome sequencing of all strains isolated for identification of each bacteriocin genes. Although the PCR technique is a general method for identification of the specific gene, it is sometimes failed to identify the gene by the mutations or the partial deletion of the target gene. In addition, whole genome sequencing provides some additional information such as the nucleotide sequencing of the flanking region of the target gene and the genome typing. By using the genome data, we found several variations of mutacin III and mutacin IV genes and designated mutacin IIIb and mutacin IVb-f. When the full-length amino acid sequence was compared between mutacin I and mutacin III/IIIb, the first 41 amino acids of mutacin I showed a 100% match with mutacin III and a 90.2% match with mutacin IIIb (Supplemental Fig. 1). Additionally, the mutacin I and mutacin III genes, with other bacteriocin-related genes, are located in the same site in chromosomal DNA^19^. We also found that the mutacin IIIb gene was located in the same site as the mutacin I and mutacin III genes in chromosomal DNA. Based on these results, mutacin IIIb is considered to be the variant of mutacin III. Moreover, mutacin III is also speculated to be a variant of mutacin I. By comparison of the antibacterial activity against various bacterial species, mutacin III- and mutacin IIIb-positive strains showed a similar antibacterial pattern against other bacteria and similar strength of their activities (Figs. 3, 4). Therefore, the biological activity of mutacin IIIb is considered to be quite similar to that of mutacin III.

In mutacin IV-positive strains, we found several variants, including 4 variants of nlmA and 3 of nlmB. Comparing single mutacin IV-positive strains, mutacin IVb-positive strains showed a similar antibacterial pattern as mutacin IVa-positive strains. However, strains with other types of bacteriocins (mutacin IVc, IVd and IVf) lost their antibacterial activities (Fig. 5). Then, we investigated the expression of the respective bacteriocin genes in mutacin IVc- and IVd-positive strains by qPCR and found low expression of the respective genes. This low expression is due to mutations in the promoter region of *nlmAB* in mutacin IVc- and mutacin IVd-positive strains. Furthermore, similar mutations of the promoter region were also found in the mutacin IVa-positive strain (KSM159) with low antibacterial activity. Therefore, we concluded that some mutacin IV-positive strains that showed low antibacterial activity were due to the low expression of the respective gene by mutation of the promoter region. Additionally, mutacin IVf-positive strains had low antibacterial activity due to the truncated *nlmB* by the insertion of one nucleotide in the *nlmB* gene. Unexpectedly, double- or triple-bacteriocin-positive strains did not show stronger activity than single-positive strains (Fig. 5b). This was due to the low expression of either or both bacteriocin genes. Although bacteriocin-producing strains possess immune factors against the respective bacteriocins, double or triple bacteriocin production may be damaging if two or three bacteriocins are highly expressed. Therefore, the mutation is suggested to be induced to protect against self-death. However, obtaining bacteriocin genes, even in the mutation or disruption of the gene coding for bacteriocin, may be an advantage to obtain immune factors because bacteriocin genes and immune factor genes are generally found in the same operon. In the expression analysis of each bacteriocin gene, we found different expression in each strain (Figs. 5, 6). In whole genome analysis, we found many mutations and deletions/insertion of the bacteriocin related genes including the genes encoding for the modification, the transport and immunity factors. The variations in these genes may be associated with the variations of the expression of each gene although we have not demonstrated.

Among 125 *S. mutans* strains, 10 strains did not possess any bacteriocin genes (mutacins I-IV, K8, Smb). However, 7 strains still showed relatively strong antibacterial activity against several oral streptococci, implying the possible presence of other bacteriocins. Previously, mutacins V and VI have also been reported to show antibacterial activity and competence^19,22,23^. We investigated the existence of these genes in all *S. mutans* strains isolated in this study by using the whole genome data and found 91 strains (72.8%) that were mutacin V-positive and 123 strains (98.4%) that were mutacin VI-positive, including 88 strains that were mutacin V- and VI- double positive. In 10 strains without bacteriocin genes (mutacins I-IV, K8, Smb), the numbers of mutacin V- and VI- double-positive, mutacin V-single positive, and mutacin VI-single positive strains were 5, 3 and 2, respectively. Seven strains with antibacterial activity showed mutacin V- and VI-double positive (5 strains), mutacin V-single positive (1 strain) and mutacin VI-single positive (1 strain) while 3 strains without antibacterial activity had both (1 strain) or either one (2 strain). Therefore, we did not find clear relation between both genes and the antibacterial activity. In addition, the results of the direct assay for mutacin I-, V- and VI-single mutants in KSM157 showed that the antibacterial activity of the mutacin I-KO mutant was significantly reduced, while the other 2 KO mutants still showed strong antibacterial activity (Fig. 8). Based on these results, we think that mutacin V or VI do not have strong antibacterial activity compared to other mutacins. In addition, we tried to identify the new bacteriocin genes by comparing the whole genome data between these 7 strains and other strains, but we could find the candidate for the specific bacteriocin genes in 7 strains. Therefore, it is speculated that the relatively strong activity of 7 strains with no major bacteriocin genes (mutacins I-IV, K8, Smb) is due to unknown bacteriocins.

In this study, we compared the antibacterial activities of each bacteriocin single-positive strain against streptococcal and nonstreptococcal species and found variations in the antibacterial pattern in each bacteriocin type. Mutacin I-, II-, III- or IIIb-positive strains showed a broad antibacterial activity, while mutacin IV-, K8- or Smb-positive strains showed strong activity against oral streptococcal species only, although the antibacterial pattern was also different among them (Figs. 4, 5). Since *S. mutans* is a key bacterium for the formation of dental plaques, the type of bacteriocin in *S. mutans* affects the composition of bacterial flora in dental plaques. To date, many investigations regarding the oral bacterial composition in saliva and dental plaque have been performed^24–28^. In particular, comparative analyses between healthy subjects and those with oral diseases, such as periodontitis, dental caries and oral cancer, have been demonstrated to identify the key bacteria or bacterial flora specific for the disease or health status. However, individual differences in bacterial composition have not been well documented. Based on our findings, we speculate that the presence of bacteriocins is one of the key factors responsible for defining the oral bacterial composition.

In conclusion, we determined the distribution of major bacteriocin genes among 125 *S. mutans* strains by analysing the whole genome sequence in each strain. We found some variations in bacteriocin genes and found that the antibacterial activity was different among bacteriocin types. Our results indicate that individual *S. mutans* strains have unique antibacterial activities affecting the bacterial composition in dental plaques.

## Materials and methods

### Bacterial strains and growth conditions

Oral streptococci, including *S*. *mutans* UA159, *S. mutans* clinical isolates, *S. mitis* GTC495*, S. gordonii* JCM12995*, S. sanguinis* GTC217*, S. parasanguinis* (clinical isolate)*, S. oralis* JCM12997, *S. salivarius* GTC215, *S. anginosus* GTC268 and *Staphylococcus aureus* MW2, were grown in trypticase soy broth (TSB) (Becton, Dickinson and Company, Franklin Lakes, NJ, USA). *Peptostreptococcus anaerobius* GTC201, *Bifidobacterium dentium* JCM1195, *Parvimonas micra* JCM12970, *Campylobacter rectus* JCM6301, *Cutibacterium acnes* JCM6425, *Actinomyces viscosus* JCM8351, and *Actinomyces israelii* IFM1905 were anaerobically grown in GAM (Nissui, Tokyo, Japan) medium at 37 °C by using Anaero Pack (Mitsubishi Gas Chemical Company Inc., Tokyo, Japan). *Corynebacterium matruchotii* JCM9386 was grown in Brain heart infusion (BHI) medium (Beckton Dickinson Microbiology Systems) at 37 °C aerobically. *Aggregatibacter actinomycetemcomitans* HK1651 was grown in TSB medium containing yeast extract (Nacalai Tesque, Kyoto, Japan; 10 g/l) (TSB-YE) at 37 °C under 5% CO_2_. The origin of all strains is listed in Supplemental Table 1.

### Isolation of *S. mutans *and *S. parasanguinis* strains

*S. mutans* strains were isolated from the oral cavity of 125 volunteers. Saliva collected from the oral cavity was plated on Mitis-Salivarius agar medium (Beckton Dickinson Microbiology Systems) containing bacitracin (final 32 μg/ml) (MSB) and incubated for 2 days at 37 °C with 5% CO_2_. The strains picked from a single colony on MSB agar and further investigated by PCR with specific primers for *S. mutans* (Supplemental Table 2). Isolated *S. mutans* strains were replated on TSB containing 2% agar (TSA) medium. The strains picked up from single colony again and finally, *S. mutans* confirmed by PCR was used in this study. Clinical isolates were designated as KSM strains. *S. mutans* isolation was approved by the ethics committee of the Kagoshima University Graduate School of Medical and Dental Sciences (No. 701) and Ethical Committee for Epidemiology of Hiroshima University (E-1998). Written informed consent was obtained from all participants. All methods were performed in accordance with the approved guidelines and regulations. *S. parasanguinis* strains was also isolated from the volunteers by using Mitis-Salivarius agar medium. Finally, the strains were verified by PCR using each specific primer (Supplemental Table 2).

### Genome sequence analysis

To perform whole genome sequencing of *S. mutans* strains, chromosomal DNA of each strain was extracted. *S. mutans* cells grown in 5 ml TSB for 12 h were collected and then suspended in 0.5 ml CS buffer (100 mM Tris–HCl [pH7.5], 150 mM NaCl, 10 mM EDTA) containing mutanolysin (Sigma-Aldrich, St. Louis, MO, USA) (5 mg/ml) and RNase (Nippon Gene, Tokyo, Japan) (10 mg/ml). After incubation at 37 °C for 2 h, proteinase K (Nacalai Tesque, Kyoto, Japan) (150 μg/ml) and SDS (final 1%) were added, followed by incubation at 55 °C for 3 h. After treatment with phenol followed by phenol–chloroform, DNA was precipitated by ethanol. Whole genome sequences (WGS) of *S. mutans* strains were obtained using the Illumina MiSeq sequencing platform, followed by annotation with Rapid Annotation using Subsystem Technology (RAST) version 2.0^29^. A phylogenetic tree was constructed using the CSI Phylogeny 1.4 pipeline available from the Center for Genomic Epidemiology (Lungby, Denmark) for SNP calling and then manipulated and annotated using the iTOL web-based tool^30^. The tree was drawn to scale, with branch lengths in the same units as those of the evolutionary distances used to infer the phylogenetic tree.

To identify bacteriocin genes in each strain, the genes encoding mutacins I-IV, K8 and Smb, which were previously identified, were searched in the National Center for Biotechnology Information (NCBI) database.

### Direct assay

To evaluate the antibacterial activity of the bacteriocins, a direct assay was performed with a previously described method^31^. Overnight culture of each *S. mutans* strain was spotted on a TSA plate and cultivated at 37 °C for 24 h. After confirming that the diameter of the growth zone of the bacteriocin-producing strain was uniformly 5 mm, 5 ml of prewarmed half-strength TSB soft agar (1%) containing indicator bacterial cells (10^7^ cells/ml) was poured over the TSA plate. The plates were incubated for 20 h under the appropriate conditions for the respective strains at 37 °C. The diameters of the growth inhibition zones surrounding the bacteriocin-producing strains were measured in three directions. Three independent experiments were performed, and the average diameter was calculated.

### Quantification of bacteriocin gene expression

Quantitative PCR was performed to investigate the expression of each bacteriocin gene. A small portion (30 µl) of overnight culture (10^8^ cells/ml) was spotted on TSA and then grown at 37 °C with 5% CO_2_ for 24 h. Bacterial cells were collected in a suspension of sterile PBS (1 ml). RNA extraction, cDNA synthesis and PCR were performed as described previously^32^.

The primers used in this study are listed in Supplemental Table 2. Finally, the gene expression was quantified against *gyrA* expression.

### Construction of bacteriocin knockout mutants

Bacteriocin deletion mutants (mutacins I, V, VI) of *S. mutans* were constructed according to a previously described method^33^. Briefly, the erythromycin resistance gene (Em^r^) with the terminator was amplified by PCR from the plasmid of pResEmNot with specific primers and cloned into pBluescript SK II (+) (yielding pBSSKEm^r^). The 5′ and 3′-flanking regions of the target gene were then amplified by PCR from *S. mutans* genomic DNA with specific primers, and each fragment was cloned into both ends of the Em^r^ gene to generate a gene cassette comprising the Em^r^ gene with the flanking region of the target gene. After PCR amplification of the whole gene, the PCR fragment was transformed into *S. mutans* with a previously described method^33^. Mutants were isolated by selection for erythromycin resistance. The primers used are listed in Supplemental Table 2.

## Supplementary information

Supplementary Information.
