# Supplementary material for: Comprehensive analysis of bacteriocins in Streptococcus mutans
Source: Sci Rep. 2021 Jun 21;11:12963. doi: 10.1038/s41598-021-92370-1 (PMC8217173; doi:10.1038/s41598-021-92370-1)
Supplement: Supplementary file 1 — Supplementary Information. [file 41598_2021_92370_MOESM1_ESM.pdf]

Comprehensive analysis of bacteriocins in *Streptococcus mutans*

Atsuko Watanabe[1], Miki Kawada-Matsuo[2,3], Mi Nguyen-Tra Le[2,3], Junzo Hisatsune[3,4], Yuichi Oogai[5], Yoshio Nakano[6], Masanobu Nakata[5], Shouichi Miyawaki[1], Motoyuki Sugai[3,4], Hitoshi Komatsuzawa[2,3]

[1]Department of Orthodontics and Dentofacial Orthopedics, Kagoshima University Graduate School of Medical and Dental Sciences, Kagoshima, Japan

[2]Department of Bacteriology, Hiroshima University Graduate School of Biomedical and Health Sciences, Hiroshima, Japan

[3]Project Research Centre for Nosocomial Infectious Diseases, Hiroshima University, Hiroshima, Japan

[4]Antimicrobial Resistance Research Centre, National Institute of Infectious Diseases, Higashi Murayama, Japan.

[5]Department of Oral Microbiology, Kagoshima University Graduate School of Medical and Dental Sciences, Kagoshima, Japan

[6] Department of Chemistry, Nihon University School of Dentistry, Tokyo, Japan

Corresponding author: Hitoshi Komatsuzawa, DDS, PhD

Department of Bacteriology, Hiroshima University Graduate School of Biomedical and Health Sciences, Kasumi 1-2-3, Hiroshima City, Hiroshima 734-8551, Japan.

Phone: +81 82 257 5635 Fax: +81 82 257 5639

E-mail: komatsuz@hiroshima-u.ac.jp

Supplemental Table 1. Strains used in this study.

Supplemental table 2. Primers used in this study.

Supplemental figure legends

Supplemental Fig. 1. Amino acid sequence of the premature peptide of bacteriocin.

Based on the genome sequence, the amino acid sequence of bacteriocin was determined. Squares represent different amino acids from those of the reference peptide of mutacin IV. NlmA-1 to 5 and nlmB 1-3 correspond to the number of nlmA and nlmB in Fig. 1.

Supplemental Fig. 2. Amino acid sequence of MutR

Based on the genome sequence, the amino acid sequence of MutR was determined in *S. mutans* strains with high (KSM126) or low (KSM22, 34, 94) levels of mutacin I expression. Squares represent different amino acids with a reference peptide.

Supplemental Table 1. Strains used in this study

strains	origin
Streptococcus mutans	
UA159	Murchison H.H. et al. Infect. Immun. 54:273-282, 1986.
KSM strains	clinical isolates
Streptococcus gordonii JCM12995	JCM collection (RIKEN BRC)
Streptococcus parasanguinis	clinical isolate
Streptococcus mitis GTC495	GTC collection (Gifu University)
Streptococcus sanguinis GTC217	GTC collection (Gifu University)
Streptococcus oralis JCM12997	JCM collection (RIKEN BRC)
Streptococcus salivarius GTC215	GTC collection (Gifu University)
Streptococcus anginosus GTC268	GTC collection (Gifu University)
Staphylococcus aureus MW2	Centers for Disease Control and Prevention (CDC). MMWR Morb Mortal Wkly Rep. 20;48(32):707-710, 1999.
Peptostreptococcus anaerobius GTC201	GTC collection (Gifu University)
Bifidobacterium dentium JCM1195	JCM collection (RIKEN BRC)
Parvimonas micra JCM12970	JCM collection (RIKEN BRC)
Campylobacter rectus JCM6301	JCM collection (RIKEN BRC)
Cutibacterium acnes JCM6425	JCM collection (RIKEN BRC)
Actinomyces viscosus JCM8351	JCM collection (RIKEN BRC)
Actinomyces israelii IFM1905	IFM collection (Chiba University)
Corynebacterium matruchotii JCM9386	JCM collection (RIKEN BRC)
Aggregatibacter actinomycetemcomitans HK1651	Haubek D et al. Int J Paediatr Dent. 16(5):370-375, 2006.

Supplemental Table 2. Primers used in this study

target gene ID	primer-forward	primer-reverse
Identification for *S. mutans* (*gtfB*)		
Smut-3368F_3481R	5’-cggtacagctcagagatgctattct-3’	5’-gccatacaccactcatgaattga-3’
Identification for *S. parasanguinis* (*fimA*)		
S.parasanguinis-F, R	5’-tggcaatgcttggttcacta-3’	5’-gatcccgttttcgagattga-3’
For quantitative PCR		
mutAI	5’-tagaagtccttggtactgaa-3’	5’-ttgaaactaggatttttcac-3’
mutII	5’-aagttaaacagtaacgcagtag-3’	5’-catgaattcatgcgacactca-3’
mutIII	5’-tcaagaagatctctttgcttttga-3’	5’-tacctgtccttgcacaacca-3’
mutIIIb	5’-gtccttggtactgaaacttttg-3’	5’-actacctgtctttgcacaacc-3’
mutIV	5’-ttgaaggtgggaaggtatcg-3’	5’-atcgaatgagtcccccaagtg-3’
mutV-1	5’-atggaattgtgcagcaggt-3’	5’-caaaggcaccagtgccaata-3’
mutV-2	5’-gtgggggatgtagttggaaa-3’	5’-tcgcaccgtatgcagctaa-3’
mutVI-1	5’-ctttcaactgttgagggtgg-3’	5’-tccagaccagcctcctaaa-3’
mutVI-2	5’-taggctttctgctgttgagg-3’	5’-ctaaagccgctccagatact-3’
Smb	5’-aagatatgttagctggggg-3’	5’-gcatcatccatgagaattg-3’
K8	5’-tgctggagttaatccaagaag-3’	5’-ggctaagaatgcccatgaat-3’
For construction of knockout strains		
mutI	5’-ataatgaaa aagcaacggt-3’	5’-cagtcgaggataatatcctcctttttcatgt-3’
	5’-gctgacctagtataaattatacttaaattgat-3’	5’-gcccaaaaaagatcaatatt-3’
mutII	5’-aaaaaaggtgatggcttac -3’	5’-cagtcgaggataataaaacacctcac-3’
	5’-gctgacctagtattaaaaattataacggg-3’	5’-acgaccgctaattcattat-3’
mutIII	5’-ataatgaaaaagcaacggt-3’	5’-cagtcgaggataatatcctcctttttcatgt-3’

mutIV	5’-tttattcgagggagtagat -3’	5’-cagtcgaggaacacccctttttcattttaa-3’
	5’-gctgacctagtttggatctgtagtttttcc -3’	5’-cctacagctaaggcgat-3’
mutV	5’-gtattcttattttacatttaaa-3’	5’- cagtcgaggatctgaaagtgcttcattatcc-3’
	5’-gctgacctagtggagctcttaattcctgt-3’	5’- gaagagagataataaaactg-3’
mutVI	5’-aacaaaaaagtttcttctgt-3’	5’- cagtcgaggattcataccaccaccctcaa-3’
	5’-gctgacctagttaggtgctgctacttttt-3’	5’- agacaacacgcattactt-3’
smb	5’-taatgatatatatgacgctc -3’	5’- cagtcgaggattttatattccttcctattctt-3’
	5’-gctgacctagttacgta ttagtgaata ttaatt-3’	5’-agttactcctttttcattta-3’
K8	5’-ttgctcgagaagaggaat-3’	5’-cagtcgaggatgcatttcgttagtagtattt-3’
	5’-gctgacctagtttagccacttgttgctca-3’	5’-ttgaatgatatcatcactct-3’

```
Mutacin I       MSNTQLLEVLGTETFDVQEDLFAFDTTDTTIVASNDDPDTRFSSLSLCSLGCTGVKNPSFNSYCC*     65
Mutacin III     MSNTQLLEVLGTETFDVQEDLFAFDTTDTTIVASNDDPDTRFKSWSLCTPGCAR--TGSFNSYCC*     63
Mutacin IIIb    MSNTQLLEVLGTETFDVQENLFTFDTTDTIVAESNDDPDTRFKSWSFCTPGCAK--TGSFNSYCC*     63
                *******************:**:****** :. *********.* *:*: **:   . ********

Mutacin II      MNKLNSNAVVSLNEVSDSELDTILGGNRWWQGVVPTVSYECRMNSWQHVFTCC*                 53


Mutacin Smb
   smbA         MKSNLLKINNVTEMEKNMVTLIKDEDMELAGGSTPACAIGVVGITVAVTGISTACTSRCINK*        62
   smbB         MKEIQKAGLQEELSILMDDANNLEQLTAGIGTTVVNSTFSIVLGNKGYICTVTVECMRNCSK*        62

Mutacin K8
   mukA1        MKNTTNEMLELIQEVSLDELDQVIGGMGKGAVGTISHECRYNSWAFLATCCS*                  52
   mukA2        MKQSDEMLELIQEVSLDELDQVIGGAGNGVIRTITQGCRMPNNMQVLFTC*                    50
   mukA3        MKQSNEMLELIQEVSLDELDQVIGGMGKGAVGTISHECRYNSWAFLATCCS*                   51

Mutacin IV
   nlmA_1a      MDTQAFEQFDVMDSQTLSTVEGGKVSGGEAVAAIGICATASAAIGGLAGATLVTPYCVGTWGLIRSH*       67
   nlmA_1b      MNTQAFEQFDVMDSQTLSTVEGGKVSGGEAVAAIGICATASAAIGGLAGATLVTPYCVGTWGLIRSH*       67
   nlmA_1c      MNTHVLEQFDVMDNETLSTVEGGKVSGGEAVAAIGICATASAAIGGLAGATLVTPYCVGTWGLIRSH*       67
   nlmA_1d      MNTHVLEQFDVMDNEILSTVEGGKVSGGEAVAAIGICATASAAIGGLAGATLVTPYCVGTWGLIRSH*       67
   nlmA_2       MNTHVLGQFDVMDNETLSTVEGGKISGGEAVAAIGICATASAAIGGLAGATLVTPYCVGTWGLIRSH*       67
   nlmA_3       MNTHVLEQFDVMDNETLSTVEGGKVSGGEAVA----------AIGGLAGATLVTPYCVGTWGLIRSH*       57
   nlmA_4       MDTQAFEQFDVMDSQTLSTVEGGKVSGGEAVA----------AIGGLVGATLVTPYCVGTWGLIRSH*       57
   nlmA_5       MNTQAFEQFDVMDNETLSTVEGGKVSGGEAVAAIGICATASAAIRGLAGATLVTPYCVGTWGLIRSH*       67
                *:*:.: ******.: ********:*******          ** **.********************


   nlmB_1a      MELNVNNYKSLTNDELSEVFGGDKQAADTFLSAVGGAASGFTYCASNGVWHPYILAGCAGVGAVGSVVFPH*   71
   nlmB_1b      MELNVNNYKSLTNGELSEVFGGDKQAADTFLSAVGGAASGFTYCASNGVWHPYILAGCAGVGAVGSVVFPH*   71
   nlmB_2       MELNVNNYKSLTNDELSEVFGGDKQAADTFLSAVGGAASGFTYCASNGVWHPYILAGCAGVGAVGSIVFPH*   71
   nlmB_3       MELNVNNYKSLTNDELLEVFGGDKQTADTFLSAVGGAASGFTYCASNGVWHPYILAGCAGVGAVGSVVFPH*   71
                *************.** ********:****************************************:*****
```

Supplemental Fig. 1. Amino acid sequence of the premature peptide of bacteriocins

```
KSM126   LKVNQSMELGELYRELRIARGLKIKDIACKNLSKSQLSRFENGQTMLAADKLLLAISGIH   60
KSM34    LKVNQSMELGELYRELRIARGLKIKDIACKNLSKSQLSRFENGQTMLAADKLLLAISGIH   60
KSM22    LKVNQSMELGELYRELRIARGLKIKDIACKNLSKSQLSRFENGQTMLAADKLLLAISGIH   60
KSM94    LKVNQSMELGELYRELRIARGLKIKDIACKNLSKSQLSRFENGQTMLAADKLLLAISGIH   60
         ************************************************************

KSM126   MSFSEFGYALSHYEESDFFKRGNKLSELYVQKDIKGLKKLLEFNDNHEVFDVYNRLNKLV   120
KSM34    MSFSEFGYALSHYEESDFFKRDNKLSELYVQKDIKGLKKLLEFNDNHEVFDVYNRLNKLV   120
KSM22    MSFSEFGYALSHYEESDFFKRGNKLSELYVQKDIKGLKKLLEFNDNHEVFDVYNRLNKLV   120
KSM94    MSFSEFGYALSHYEESDFFKRGNKLSELYVQKDIKGLKKLLEFNDNHEVFDVYNRLNKLV   120
         *********************.**************************************

KSM126   IQVTIHLLDTDYIISDDDKNFLTTYLYNIEEWTEYELYIFGNTMSILSSDDLIFLGKAFV   180
KSM34    IQVTIHLLDTDYIISDDDKNFLTTYLYNIEEWTEYELYIFGNTMSILSSDDLIFLGKAFV   180
KSM22    IQVTIHLLDTDYIISDDDKNFLTTYLYNIEEWTEYELYIFGNTMSILSSDDLIFLGKAFV   180
KSM94    IQVTIHLLDTDYIISDDDKNFLTTYLYNIEEWTEYELYIFGNTMSILSSDDLIFL*KAFV   179
         ******************************************************* ****

KSM126   ERDKLYISLPSHKKNAELTFLNLILILLERKKLYQAIYFVENLEKLLNYQDMFAITFLKF   240
KSM34    ERDKLYISLPSHKKNAELTFLNLILILLERKKLYQAIYFVENLEKLLNYQDMFAITFLKF   240
KSM22    ERDKLYISLPSHKKNAELTFLNLILILLERKKLYQAIYIVENLEKLLNYQDMFAITFLKF   240
KSM94    ERDKLYISLPSHKKNAELTFLNLILILLERKKLYQAIYFVENLEKLLNYQDMFAITFLKF   239
         **************************************:*********************

KSM126   LKKIITYFHDKSVDMSELEHYINIVEEINPTIASILKSNLNQLLSSFSH*   289
KSM34    LKKIITYFHDKSVDMSELEHYINIVEEINPTIASILKSNLNQLLSSFSH*   289
KSM22    LKKIITYFHDKSVDMSELEHYINIVEEINPTIASILKSNLNQLLSSFSH*   289
KSM94    LKKIITYFHDKSVDMSELEHYINIVEEINPTIASILKSNLNQLLSSFSH*   288
         *************************************************
```

Supplemental Fig. 2. Amino acid sequence of MutR
